# Comparing person-level matching algorithms to identify risk across disparate datasets among patients with a controlled substance prescription: retrospective analysis

**DOI:** 10.1093/jamiaopen/ooac020

**Published:** 2022-03-30

**Authors:** Lindsey M Ferris, Jonathan P Weiner, Brendan Saloner, Hadi Kharrazi

**Affiliations:** 1 Department of Health Policy and Management, Johns Hopkins Bloomberg School of Public Health, Baltimore, Maryland, USA; 2 The Chesapeake Regional Information System for our Patients, Baltimore, Maryland, USA; 3 Johns Hopkins Center for Population Health Information Technology, Johns Hopkins Bloomberg School of Public Health, Baltimore, Maryland, USA

**Keywords:** analgesics, opioid, overdose, medical record linkage, databases, factual, public health

## Abstract

**Background:**

The opioid epidemic in the United States has precipitated a need for public health agencies to better understand risk factors associated with fatal overdoses. Matching person-level information stored in public health, medical, and human services datasets can enhance the understanding of opioid overdose risk factors and interventions.

**Objective:**

This study compares approximate match versus exact match algorithms to link disparate datasets together for identifying persons at risk from an applied perspective.

**Methods:**

This study used statewide prescription drug monitoring program (PDMP), arrest, and mortality data matched at the person-level using an approximate match and 2 exact match algorithms. Impact of matching was assessed by analyzing 3 independent concepts: (1) the prevalence of key risk indicators used by PDMP programs in practice, (2) the prevalence of arrests and fatal opioid overdose, and (3) the performance of a multivariate logistic regression for fatal opioid overdose. The PDMP key risk indicators included (1) multiple provider episodes (MPE), or patients with prescriptions from multiple prescribers and dispensers, (2) high morphine milligram equivalents (MMEs), which represents an opioid’s potency relative to morphine, and (3) overlapping opioid and benzodiazepine prescriptions.

**Results:**

Prevalence of PDMP-based risk indicators were higher in the approximate match population for MPEs (*n* = 4893/1 859 445 [0.26%]) and overlapping opioid/benzodiazepines (*n* = 57 888/1 859 445 [4.71%]), but the exact-basic match population had the highest prevalence of individuals with high MMEs (*n* = 664/1 910 741 [3.11%]). Prevalence of arrests and deaths were highest for the approximate match population compared with the exact match populations. Model performance was comparable across the 3 matching algorithms (exact-basic validation area under the receiver operating characteristic curve [AUC]: 0.854; approximate validation AUC: 0.847; exact + zip validation AUC: 0.826) but resulted in different cutoff points balancing sensitivity and specificity.

**Conclusions:**

Our study illustrates the specific tradeoffs of different matching methods. Further research should be performed to compare matching algorithms and its impact on the prevalence of key risk indicators in an applied setting that can improve understanding of risk within a population.

## Lay Summary

The persistence of the opioid epidemic necessitates an understanding of risk factors for fatal opioid overdose across multiple sources of data. However, without a national identifier for individuals that can be used to link the data from multiple sources together, the data are linked together using personal identifiers in a variety of ways. This study combines prescription histories, criminal justice encounters, and deaths using several different approaches to linking the data together at the person level to provide insight into the impact on understanding risk of fatal opioid overdose. Several risk indicators, prevalence of arrests and deaths, and models predicting risk were quantified for 3 approaches to linking being used in practice. As more studies call for combining cross-sector datasets for more comprehensive analysis, state programs must understand the implications of the data linking process when designing public health programs and interventions that benefit the community.

## INTRODUCTION

Individuals at risk of opioid-related overdose often interact with multiple service systems, including healthcare, public health, social, and human service agencies. As individuals interact with each domain, information about their complex needs, characteristics, and service provisions are recorded in electronic databases. Although the ease of matching electronic data has improved for single datasets, matching person-level data across distinct agencies remains a major impediment to dataset linkage.^1^ Most datasets remain siloed without a common identifier to efficiently match separate person-level datasets to support a more comprehensive understanding of an individual’s risk of overdose.^2^ Populations at risk of overdose are not homogenous, but rather likely to be only partially represented in clinical, criminal justice, and other data. If linked, analyses using an integrated database that captures a more comprehensive cross-section of a patient’s indicators related to opioid misuse or addiction could improve the understanding and identification of individuals at risk for opioid-related overdose and other negative outcomes that may not be possible if using a single source of data.

Absent a nationwide unique identifier for patients, alternative analytic techniques are being used to match person-level data together from different sources using personal demographics and identifiers. Most commonly, “exact” and/or “approximate” matching algorithms are utilized.^3^ Exact matching relies on comparing a set of identifiers (eg, name, age, sex, etc.) and determining a match when those identifiers match exactly (eg, persons with the same name and date of birth [DOB] in 2 databases), while approximate matching uses a weighted analytic algorithm applied to patient identifiers to derive a score that determines whether a certain matching threshold was reached.^3^ These 2 approaches, exact and approximate matching, are being used in practice today to bring disparate datasets together.

Approximate matching is a fairly common applied technique within healthcare, particularly within health information exchanges (HIEs) and large multi-system health organizations.^4^ Approximate algorithms have been found to have a higher degree of matching accuracy and a strong potential to link individuals across datasets without a common identifier by accommodating discrepancies (eg, nicknames, transposed digits, changes in surname) in the demographic variables than exact match algorithms.^5^^,^^6^ A handful of studies examining opioid overdose outcomes have matched cross-domain datasets (eg, electronic health records, prescription drug monitoring program [PDMP], and deaths) together with public domain software applying both exact match and approximate match algorithms.^7^^,^^8^

Despite the improved performance, access to approximate matching is not always available and exact matching must be used.^9–13^ Exact match on the individual’s name and DOB are most commonly used, with some studies also using sex, county of residence, and social security number as additional matching criteria.^9–13^ One large-scale example of combining data from multiple agencies was a statewide opioid overdose analysis performed by Massachusetts’ state government that linked fifteen datasets together using a series of exact match algorithms.^14^ As matching cross-sector datasets to understand risks related to opioids becomes more common among state PDMPs, Departments of Health, and other state and local programs, additional research needs to be done to understand the impact of matching techniques.

A recent analysis examined the impact of an exact match algorithm against a proprietary approximate (probabilistic) algorithm on the prevalence of key high-risk indicators within PDMP data, and demonstrated that the degree of the impact varied by measure.^15^ This study, using statewide Maryland data, builds on these concepts by comparing 2 exact matching algorithms with an approximate matching algorithm, all of which are being used in practice today, with aim of quantifying the relative effect the record linkage approaches on several independent concepts: (1) the prevalence of patient risk indicators using PDMP data, (2) the prevalence of arrests and deaths among patients with PDMP data, and (3) the performance of a risk model for fatal opioid overdose.

## METHODS

### Study design, population, and data sources

A retrospective cohort analysis began with 2015 Maryland PDMP data and included individuals with one or more prescriptions. The MD PDMP collects schedule II-V controlled substances (ie, opioids, sedatives, stimulants, and other drugs for medicinal use with potential for abuse) dispensed to Maryland residents by pharmacists, dispensing prescribers, and mail-order pharmacies. The PDMP data from dispenser information systems were centrally collected by a statewide vendor’s software that has its own native matching algorithm to determine unique identities (totaling 3 304 446 in 2015) prior to being processed by the approximate or exact match algorithms. Starting with the vendor-defined identities, the matching algorithms were applied to the PDMP data such that individuals were matched within the dataset before being matched with external datasets. This resulted in the creation of a new unique master identifier for every identity included in the study specific to each matching algorithm.

After applying the matching algorithms to the PDMP database, matching was performed across the arrest and mortality data. Individuals with property- or drug-related arrests between 2013 and 2015 from the Maryland Department of Public Health Safety and Correctional Services (DPSCS) were matched with individuals in the PDMP data. Criminal justice involvement is relevant to future opioid-related outcomes and 3 years of data were included to ensure a high enough sample size.^16^ DPSCS uses a State Identification Number to positively identify unique individuals within their native system using the arrestees fingerprints. Deaths from 2015 to 2016 were matched with the PDMP data, with the outcome-of-interest of opioid-related overdose deaths. Mortality data for investigated deaths were provided by the Office of the Chief Medical Examiner (OCME) and contained identifying information for the decedent, date of death, and cause of death for all drug- and alcohol-related overdose deaths in MD. The final limited dataset for research contained only the unique identifiers and IRB-approved variables for analysis. IRB approval was obtained from the Johns Hopkins Bloomberg School of Public Health and the Maryland Department of Health (IRB #00007542). Supplementary File S1 depicts a graphical representation of the final dataset.

### Model variables

The target outcome of fatal opioid overdose was defined as having a cause-of-death indicator in the OCME dataset for illicit or licit opioids, including any of the following substances: prescription opioids, hydrocodone, hydromorphone, methadone, morphine, oxycodone, oxymorphone, tramadol, heroin, or fentanyl. Intentional, unintentional, and undetermined intent were all included.

Model variables were derived from the PDMP data based on common risk indicators found in the literature or established as national clinical quality improvement outcome measures.^7^^,^^10^^,^^17–19^ Model variables included sex, age group, method of payment for prescriptions (modal), number of opioid prescribers and dispensers, and prescriptions for methadone, long-acting opioids, buprenorphine opioids, shorting-acting schedule II opioids, short-acting schedule III/IV opioids, benzodiazepines, other nonbenzodiazepine sedatives, and muscle relaxants.

### Arrest, death, and PDMP-based variables

We considered four independent markers that are associated with high risk of overdose using PDMP data.^20^ Prevalence of several complex variables based on thresholds most commonly used by PDMP programs to identify high-risk individuals within a rolling 3-month window for the duration of the study period was also analyzed^20^: (1) multiple provider episodes (MPEs), defined as 5 unique prescribers and 5 unique dispensers for all controlled substances; (2) high daily average morphine milligram equivalents (MMEs), defined as ≥ 90 mg/day average daily dose and ≥ 60 days’ supply opioids; and (3) overlapping opioid and benzodiazepine prescriptions, where overlap occurs for 25% or more of the days’ supply (for days’ supply > 5 days) if the patient had ≥ 60 days’ supply opioids. The variables were analyzed separately to mimic the approach PDMP programs take when evaluating high-risk individuals.

Variables were also constructed for any individual with at least one arrest and any individual who experienced a fatal opioid overdose.

### Person-level matching techniques

Prior to removing personal identifiers from the research database, the datasets were matched using an approximate match algorithm and 2 different exact match algorithms. The matching algorithms relied on all or a subset of identifiable personal and demographic data available, described in more detail below.

#### Approximate match linkage

The approximate match algorithm used in this study was the master patient index (MPI) technology (IBM InfoSphere^®^, v10.1) deployed and operated since 2010 by Maryland’s state-designated, nonprofit HIE, CRISP (Chesapeake Regional Information System for our Patients). The algorithm compares each of the demographic data elements (using an advanced approach of grouping multiple attributes together into more unique combinations for fast comparison), assigns a score to each comparison, then tallies up to a final score to determine matching. If the final score passes the CRISP-defined threshold for a match, the records are considered part of the same master identity and are matched together. Records that did not meet the threshold remain as separate identities. The demographics leveraged by the algorithm include: first name, last name, DOB, gender, address, phone number, and social security number (if available).

#### Exact match linkage

Two levels of exact match algorithms were applied to the data based on availability of the demographic elements, common approaches in published literature,^11^^,^^14^ and what is being used in practice by state-based programs attempting to bring multiple datasets together operationally to understand opioid risk today. The first exact match algorithm (“exact-basic”) used an exact match on first name, last name, gender, and DOB. The second exact match algorithm (“exact + zip”) used an exact match on the same elements as the first algorithm (name, gender, and DOB), plus ZIP Code. Adding ZIP code presumably provides more conservative and “accurate” matching among individuals but will not accommodate transient or purposely evasive individuals. Gender was normalized to male, female, or unknown. Minor adjustments to first name were made to ensure no middle names or initials were included in the first name field. To reflect current practice, no close-match, near-match, or phonetic matching logic was applied. The PDMP dataset was processed first by comparing identities within the dataset and creating a new master identifier for any matched identities. Next, the identities in the arrest and death files were compared with the identities in the PDMP dataset. If multiple records within a single database had matching demographics, the master identifier would be applied across all records, therefore matching records within a single database as well as across databases. This process was repeated for both exact match algorithms, resulting in 2 separate sets of exact match master identifiers. See Supplementary Files S2 and S3 for further details of all 3 matching algorithms.

### Statistical analysis

Each matching algorithm requires the demographic data to be at a high enough quality level to ensure sufficient matching. Prior to data linkage, the demographic variables used for matching in each dataset were assessed for completeness (number of occurrences of missing values for each data field).^21^ Postlinkage, the characteristics of the population identified by the different matching algorithms were described. A multivariate logistic regression analysis for risk of fatal opioid overdose was performed on the population defined by each matching algorithm using split-half technique (60% development, 40% validation using random selection) to compare model performance when different patient matching algorithms are leveraged for the same population.^22^ Model performance was measured using sensitivity, specificity, and area under the receiver operating characteristic curve (AUC), measuring the ability of the model to discriminate between individuals truly at risk (sensitivity) from individuals truly not at risk (specificity), ranging from 0 to 1. The optimal cutoff point for the model, which maximizes the sensitivity and specificity, was compared across the 3 matching algorithms. Finally, the prevalence of unique individuals with a PDMP-based high-risk indicator, an arrest, or an opioid-related overdose death and death rates per 1000 were calculated for the population matched by each algorithm.

## RESULTS

### Quality of matching fields

All datasets contained the common matching fields (ie, name, DOB, sex, address, city, state, and zip) with high degrees of completeness between 93.8% and 100% (Supplementary File S4). The PDMP and death files had no Social Security Number’s (SSN’s) available for matching and arrest file had 61.0% completeness. Thus, SSN was only taken into account by the approximate match algorithm, which is inherently designed to leverage SSN for matching when supplied, but could not be used for the exact match algorithms. Although the address fields were well-populated (completeness between 95.9% and 100%), they were not standardized in any dataset, limiting the potential for exact matches, and was also therefore only leveraged by the approximate algorithm.

### Study population

Using the approximate match algorithm, a total of 1 859 445 individuals were identified within the PDMP dataset, of which 1318 (0.07%) individuals experienced a fatal opioid overdose and 8712 (0.47%) had an arrest record. The exact-basic algorithm resulted in a total of 1 910 741 individuals (2.8% more identities than approximate matching), of which, 1167 (0.06%) fatally overdosed and 8589 (0.45%) had an arrest record. The exact + zip algorithm resulted in a total of 2 065 019 individuals (11.1% more identities than approximate matching), of which, 605 (0.03%) fatally overdosed and 3839 (0.19%) had an arrest record (Table 1). The full population’s characteristics were consistent across the 3 matching methods; however, differences were more pronounced in the death cohorts. The exact-basic death cohort had 2.98% more males and 2.42% more prescriptions with Medicaid as a method of payment and the exact + zip death cohort had 3.58% more individuals aged 50–64 years and 3.31% more Self-Pay prescriptions than the approximate match population. One of the most distinguishable differences between the exact and approximate match algorithms was the ≥3 opioid prescribers (exact-basic: −2.81%; exact + zip: −7.57%) and ≥3 opioid dispensers (exact-basic: −2.54%; exact + zip: −7.93%) variables.

**Table 1.** Characteristics of study population for each matching algorithm^a^

Characteristic, *n* (%)	Approximate cohort		Exact-basic algorithm cohort^b^				Exact + zip algorithm cohort^b^			
Death status	Full (*n* = 1 859 445)	Deaths (*n* = 1318)	Full (*n* = 1 910 741)	% Difference^c^	Deaths (*n* = 1167)	% Difference^c^	Full (*n* = 2 065 019)	% Difference^c^	Deaths (*n* = 605)	% Difference^c^
Male	775 716 (41.72)	849 (64.37)	794 564 (41.61)	−0.09	788 (67.35)	2.98	856 100 (41.47)	−0.23	393 (64.96)	0.59
Age group (years)										
Under 18	140 648 (7.56)	0 (0)	142 288 (7.45)	−0.11	0 (0)	0.00	152 207 (7.37)	−0.19	0 (0)	0.00
18–34	410 834 (22.09)	378 (28.68)	418 770 (21.93)	−0.16	338 (28.89)	0.23	461 323 (22.35)	0.26	165 (27.27)	−1.41
35–49	427 737 (23.00)	488 (37.03)	440 897 (23.09)	0.09	437 (37.35)	0.35	485 742 (23.53)	0.53	212 (35.04)	−1.99
50–64	520 899 (28.01)	419 (31.79)	537 772 (28.16)	0.15	370 (31.62)	−0.08	579 104 (28.05)	0.04	214 (35.37)	3.58
Over 65	359 327 (19.33)	33 (2.50)	369 856 (19.37)	0.04	25 (2.14)	−0.36	386 042 (18.70)	−0.63	14 (2.31)	−0.19
Method of Payment										
Self Pay	291 474 (15.68)	135 (10.24)	302 575 (15.85)	0.17	125 (10.68)	0.44	320 322 (15.52)	−0.16	82 (13.55)	3.31
Medicaid	268 537 (14.44)	475 (36.04)	271 363 (14.21)	−0.23	450 (38.46)	2.42	311 520 (15.09)	0.65	213 (35.21)	−0.83
Medicare	150 139 (8.07)	123 (9.26)	154 659 (8.10)	0.03	99 (8.46)	−0.80	165 832 (8.03)	−0.04	59 (9.75)	0.49
Commercial	1 103 135 (59.33)	559 (42.41)	1 131 679 (59.26)	−0.07	484 (41.37)	−1.04	1 213 700 (58.79)	−0.54	246 (40.66)	−1.75
Military/VA	10 673 (0.57)	18 (1.37)	11 599 (0.61)	0.04	4 (0.34)	−1.03	12 392 (0.60)	0.03	2 (0.33)	−1.04
Workers Comp	9 383 (0.50)	2 (0.15)	10 443 (0.55)	0.05	3 (0.26)	0.11	11 232 (0.54)	0.04	3 (0.50)	0.35
Unknown/Other	26 104 (1.40)	7 (0.53)	27 265 (1.43)	0.03	5 (0.43)	−0.10	29 420 (1.43)	0.03	0 (0)	−0.53
Medication										
Opioid prescribers ≥3	172 105 (9.26)	420 (31.87)	171 963 (9.00)	−0.26	340 (29.06)	−2.81	164 913 (7.99)	−1.27	147 (24.30)	−7.57
Opioid dispensers ≥3	78 961 (4.25)	305 (23.14)	74 073 (3.88)	−0.37	241 (20.60)	−2.54	58 138 (2.82)	−1.43	92 (15.21)	−7.93
Methadone fills ≥1	10 194 (0.55)	57 (4.32)	10 606 (0.56)	0.01	46 (3.93)	0.39	12 069 (0.58)	0.03	24 (3.97)	−0.35
Opioid LA fills ≥1	70 589 (3.80)	190 (14.42)	73 696 (3.86)	0.06	158 (13.50)	−0.92	80 657 (3.91)	0.11	83 (13.72)	−0.70
Opioid OUD fills ≥1	28 339 (1.52)	200 (15.17)	28 453 (1.49)	−0.03	181 (15.47)	0.30	34 326 (1.66)	0.14	78 (12.89)	−2.28
Opioid SA-2 fills ≥4	885 205 (47.61)	877 (66.46)	908 770 (47.56)	−0.05	772 (65.98)	−0.48	968 287 (46.89)	−0.72	396 (65.45)	−1.01
Opioid other SA-3,4 fills ≥1	458 851 (24.68)	376 (28.53)	465 086 (24.34)	−0.34	315 (26.92)	−1.61	479 594 (23.22)	−1.46	143 (23.64)	−4.89
Benzodiazepine fills ≥2	463 008 (24.90)	639 (48.48)	471 180 (24.66)	−0.24	551 (47.09)	−1.39	500 000 (24.21)	−0.69	285 (47.11)	−1.37
Muscle relaxant fills ≥1	19 300 (1.04)	65 (4.93)	19 789 (1.04)	−0.00	57 (4.87)	−0.06	21 295 (1.03)	−0.01	31 (5.12)	0.19
Sedative fills ≥1	138 643 (7.46)	187 (14.19)	141 174 (7.39)	−0.07	150 (12.82)	−1.37	148 940 (7.21)	−0.25	79 (13.06)	−1.13
High MME (≥90 mg/day)	57 314 (3.08)	226 (17.15)	59 423 (3.11)	0.03	178 (15.21)	−1.94	63 454 (3.07)	−0.01	95 (15.70)	−1.45
Overlapping opioid/benzo	87 805 (4.72)	311 (23.60)	88 373 (4.63)	−0.09	244 (20.85)	−0.09	90 476 (4.38)	−0.34	126 (20.83)	−2.77
Legal										
Has any arrest	8825 (0.47)	113 (8.57)	8589 (0.45)	−0.02	107 (9.15)	0.58	3 839 (0.19)	−0.28	27 (4.46)	−4.11

All boldface numbers indicate significance at the *P* < .001 level. Benzo: benzodiazepine; LA: long-acting; MME: morphine milligram equivalent; OUD: opioid use disorder (buprenorphine); SA: short-acting; VA: Veteran’s Affairs.

Population consists of drug and property arrests from 2013 to 2015, PDMP data from 2015, and an outcome of fatal opioid overdose in 2015 or 2016.

Exact-basic algorithm matched first name, last name, gender, date of birth. Exact + zip algorithm matched first name, last name, gender, date of birth, and ZIP code.

% difference is the approximate algorithm minus the exact algorithm percentage for the full and death cohorts.

### Statistical analysis

The statistically significant predictors in the fatal opioid overdose risk model were relatively consistent between the approximate match algorithm and the exact match algorithms, with a few exceptions (Table 2). Self-pay was a predictor for the model run on the exact-basic match (odds ratio [OR], 1.39; 95% confidence interval [CI], 1.08–1.78) and the exact + zip match (OR, 1.64; 95% CI, 1.19–2.27) populations but not the approximate match population. High MME was a predictor for the approximate match (OR, 1.36; 95% CI, 1.02–1.80) and exact + zip (OR, 1.75; 95% CI, 1.15–2.68) populations, but not the exact-basic population. Finally, ≥3 opioid prescribers and ≥1 methadone fill variables were not statistically significant predictors for the exact + zip population, despite being a predictor for the approximate and exact-basic populations.

**Table 2.** Odds ratios and bias for populations matched by each matching algorithm^a^

Characteristic	Approximate (*N* = 1 859 445)		Exact-basic algorithm (*N* = 1 910 753)^b^			Exact + zip algorithm (*N* = 2 065 023)^b^		
	*n*	OR (95% CI)	*n*	OR (95% CI)	Bias^c^	*n*	OR (95% CI)	Bias^c^
Male	774 868 (41.70)	**2.86 (2.45–3.32)**	794 564 (41.61)	**2.85 (2.44–3.34)**	0.0	856 100 (41.47)	**2.90 (2.34–3.60)**	−1.4
Age group (years)								
Under 18	140 648 (7.57)	—	142 288 (7.45)	—		152 207 (7.37)	—	
18–34	410 456 (24.83)	Reference	418 770 (21.93)	Reference		461 323 (22.35)	Reference	
35–49	427 249 (25.85)	1.01 (0.85–1.21)	440 897 (23.09)	1.04 (0.87–1.25)	−289.6	485 742 (23.53)	1.02 (0.79–1.32)	−64.2
50–64	520 480 (31.49)	**0.69 (0.58–0.84)**	537 772 (28.16)	**0.77 (0.63–0.93)**	26.0	**579** **104 (28.05)**	0.82 (0.63–1.07)	45.8
65–80	294 821 (17.84)	**0.09 (0.06–0.16)**	303 760 (15.91)	**0.07 (0.04–0.13)**	−12.0	**317** **083 (15.36)**	**0.06 (0.02–0.14)**	−22.7
Over 80	64 473 (3.47)	—	66 096 (3.46)	—		68 959 (3.34)	—	
Method of Payment								
Self Pay	291 339 (15.68)	1.23 (0.96–1.57)	302 575 (15.85)	**1.39 (1.08–1.78)**	−60.6	320 322 (15.52)	**1.64 (1.19–2.27)**	−144.9
Medicaid	268 062 (14.43)	**2.55 (2.15–3.01)**	271 363 (14.21)	**2.99 (2.51–3.54)**	−17.1	311 520 (15.09)	**2.68 (2.11–3.41)**	−5.7
Medicare	150 017 (8.07)	**2.46 (1.87–3.23)**	154 659 (8.10)	**2.70 (2.03–3.58)**	−10.2	165 832 (8.03)	**2.92 (2.01–4.24)**	−19.1
Commercial	1 102 576 (59.34)	Reference	1 131 679 (59.26)	Reference		1 213 700 (58.79)	Reference	
Military/VA	10 655 (0.57)	**3.25 (1.81–5.81)**	11 599 (0.61)	0.66 (0.17–2.68)	193.6	12 392 (0.60)	0.60 (0.08–4.27)	144.0
Workers Comp	9 381 (0.50)	0.28 (0.04–2.01)	10 443 (0.55)	0.59 (0.15–2.39)	58.7	11 232 (0.54)	1.01 (0.25–4.11)	101.1
Unknown/Other	26 097 (1.40)	0.28 (0.07–1.13)	27 265 (1.43)	0.45 (0.14–1.41)	37.4	29 420 (1.43)	—	100.0
Medication								
Opioid prescribers ≥3	171 685 (9.24)	**1.53 (1.23–1.91)**	171 963 (9.00)	**1.47 (1.17–1.85)**	10.0	164 913 (7.99)	1.31 (0.95–1.81)	36.2
Opioid dispensers ≥3	78 656 (4.23)	**1.83 (1.45–2.30)**	74 073 (3.88)	**1.57 (1.24–2.00)**	24.9	58 138 (2.82)	**1.56 (1.09–2.23)**	26.4
Methadone fills ≥1	10 137 (0.55)	**2.05 (1.40–3.00)**	10 606 (0.56)	**2.04 (1.36–3.08)**	0.0	12 069 (0.58)	1.57 (0.86–2.89)	36.6
Opioid Long-Acting fills ≥1	70 399 (3.79)	1.06 (0.80–1.39)	73 696 (3.86)	1.36 (0.94–1.68)	−333.1	80 657 (3.91)	1.27 (0.85–1.90)	−343.4
Opioid OUD fills ≥1	28 139 (1.51)	**4.88 (3.93–6.04)**	28 453 (1.49)	**5.10 (4.09–6.37)**	−2.1	34 326 (1.66)	**5.58 (4.07–7.65)**	−8.5
Opioid SA-2 fills ≥4	884 329 (47.59)	**1.38 (1.16–1.65)**	908 770 (47.56)	**1.63 (1.36–1.96)**	−52.2	968 287 (46.89)	**1.59 (1.24–2.04)**	−43.2
Opioid other SA-3,4 fills ≥1	458 475 (24.67)	1.03 (0.87–1.23)	465 086 (24.34)	1.05 (0.88–1.26)	−61.4	479 594 (23.22)	1.16 (0.90–1.50)	−344.5
Benzodiazepine fills ≥2	462 369 (24.88)	**2.08 (1.74–2.48)**	471 180 (24.660	**2.34 (1.96–2.80)**	−16.7	500 000 (24.21)	**2.24 (1.74–2.88)**	−10.3
Muscle relaxant fills ≥1	19 235 (1.04)	**1.49 (1.04–2.14)**	19 789 (1.04)	**2.05 (1.46–2.89)**	−79.5	21 295 (1.03)	**2.43 (1.55–3.81)**	−121.6
Sedative fills ≥1	138 456 (7.45)	**1.69 (1.38–2.08)**	141 174 (7.39)	**1.59 (1.27–1.97)**	12.3	148 940 (7.21)	**1.57 (1.15–2.14)**	15.0
High MME	57 088 (3.07)	**1.36 (1.02–1.80)**	59 423 (3.11)	1.22 (0.90–1.66)	33.3	63 454 (3.07)	**1.75 (1.15–2.68)**	−84.5
Overlapping opioid/benzo	87 494 (4.71)	**1.57 (1.23–2.01)**	88 373 (4.63)	1.21 (0.94–1.56)	58.2	90 476 (4.38)	1.15 (0.79–1.67)	69.5
Legal								
Has any arrest	8 712 (0.47)	**4.59 (3.52–6.00)**	8 589 (0.45)	**4.58 (3.46–6.06)**	−0.1	3 839 (0.19)	**5.01 (2.86–8.76)**	−5.6

Benzo: benzodiazepine; CI: confidence interval; MME: morphine milligram equivalent; OR: odds ratio; OUD: opioid use disorder (buprenorphine); SA: short-acting; VA: Veteran’s Affairs.

Population consists of drug and property arrests from 2013 to 2015, PDMP data from 2015, and an outcome of fatal opioid overdose in 2015 or 2016.

Exact-basic algorithm matched first name, last name, gender, and date of birth. Exact + zip algorithm matched first name, last name, gender, date of birth, and ZIP code.

Bias refers to the difference in log odds coefficients in each multivariable model, compared with the approximate using the equation: 100*[(logit_reference—_logit_comparison_/logit_reference_)].

Although the performance of the model was comparable across the 3 matching algorithms (exact-basic validation AUC: 0.854; approximate validation AUC: 0.847; exact + zip validation AUC: 0.826), there were different optimal cutoff points balancing sensitivity and specificity (Table 3). The cutoff point for the approximate match algorithm was 0.0010 (sensitivity: 67.54%; specificity: 84.29%), resulting in a total of 104 293 high-risk individuals, of which, 362 died from a fatal opioid overdose (3.47 deaths per 1000 high-risk individuals). This is in comparison with the exact-basic algorithm, which had a cutoff point of 0.0005 (sensitivity: 87.47%, specificity: 66.26%), resulting in 229 646 high-risk individuals and 384 deaths (1.67 deaths per 1000 high-risk individuals), and the exact + zip algorithm, which had a cutoff point of 0.00025 (sensitivity: 85.53%, specificity: 62.17%), resulting in 275 352 high-risk individuals and 195 deaths (0.71 deaths per 1000 high-risk individuals). See Supplementary File S5 for classification tables of each model run on the population linked by the 3 matching methods.

**Table 3.** Model performance for opioid overdose death for populations matched by each algorithm^a^

Model performance	Approximate algorithm (*N* = 1 859 445)	Exact-basic algorithm(*N* = 1 910 741)^b^	Exact + zip algorithm(*N* = 2 065 019)^c^
Optimal cutoff point	0.0010	0.0005	0.00025
Derivation AUC	0.858	0.860	0.837
Validation AUC	0.847	0.854	0.826
Sensitivity	67.54	87.47	60.96
Specificity	84.29	66.26	42.54
# of high-risk patients	104 293	229 646	275 352
% of validation cohort	15.8	33.78	37.85
# of deaths among high-risk patients	362	385	195
Deaths per 1000 high risk patients	3.47	1.67	0.71

AUC: area under the curve.

Population consists of drug and property arrests from 2013 to 2015, PDMP data from 2015, and an outcome of fatal opioid overdose in 2015 or 2016.

Exact-basic algorithm matched first name, last name, gender, and date of birth.

Exact + zip algorithm matched first name, last name, gender, date of birth, and zip code.

### Arrest, death, and PDMP-based risk indicators and death rate statistics

Examining the prevalence of PDMP-based risk indicators, arrests, and deaths identified by each of the algorithms further demonstrates the impact the matching can have on understanding patient-level risk (Table 4). Prevalence of PDMP-based risk factors was highest within the approximate match population for the MPE (*n* = 4893/1 859 445 [0.26%]) and overlapping opioid and benzodiazepine (*n* = 57 888/1 859 445 [4.71%]) PDMP-based risk measures, but the exact-basic match population yielded the highest prevalence of individuals with high MME (*n* = 664/1 910 741 [3.11%]). Prevalence of individuals with an arrest and death were highest for the approximate match population (arrest: *n* = 8812/1 859 445 [0.47%]; opioid overdose death: *n* = 1318/1 859 445 [0.07%]) compared with the exact-basic (arrest: *n* = 8589/1 910 741 [0.45%]; opioid overdose death: *n* = 1167/1 910 741 [0.06%]) and exact + zip (arrest: *n* = 3839/2 065 019 [0.19%]; opioid overdose death: *n* = 605/2 065 019 [0.03%]) match populations.

**Table 4.** Risk indicator prevalence for individuals identified by each matching algorithm

High risk indicators/outcome	Identified by approximate algorithm (*n* = 1 859 445)	Identified by exact-basic algorithm (*n* = 1 910 741)^a^	Identified by exact + zip algorithm (*n* = 2 065 019)^b^
MPE	4893 (0.26)	4443 (0.23)	2552 (0.12)
High MME	57 088 (3.07)	59 423 (3.11)	63 454 (3.07)
Overlapping opioid/benzo	87 494 (4.71)	88 373 (4.63)	90 476 (4.38)
Arrest	8812 (0.47)	8589 (0.45)	3839 (0.19)
opioid overdose death	1318 (0.07)	1167 (0.06)	605 (0.03)

*Note*: All Chi-squared tests were significant at the *P* < .001 level

Benzo: benzodiazepine; MME: morphine milligram equivalents; MPE: multiple provider episode.

Exact-basic algorithm matched first name, last name, gender, and date of birth.

Exact + zip algorithm matched first name, last name, gender, date of birth, and zip code.

**Figure 1.**
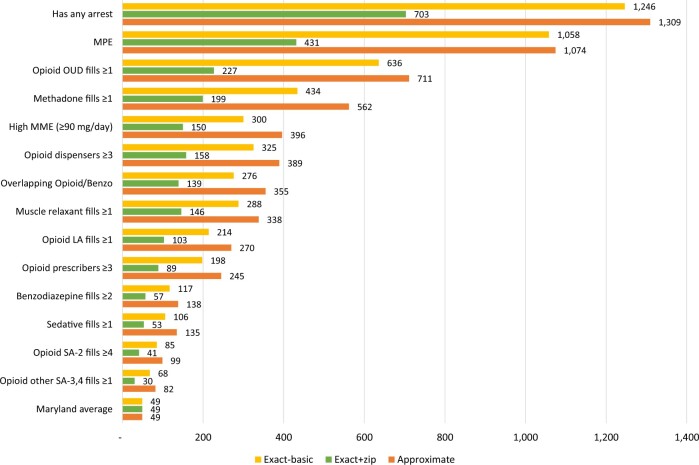
Death rates (per 100 000) for individuals with a risk factor

Finally, deaths per 100 000 in the denominator were calculated for variables included in the multivariable model (Figure 1). The population linked using the approximate match algorithm universally resulted in capturing the highest death rates per predictor as compared with the exact match algorithms. The highest deaths per 100 000 involved individuals who had any arrest (approximate: 1309; exact-basic: 1246; exact + zip: 703) or individuals with MPEs (approximate: 1074; exact-basic: 1058, exact + zip: 431). All predictors for the population linked via the approximate and exact-basic algorithms had a higher death rate than the Maryland average (49 per 100 000). The exact + zip algorithm had 2 predictors lower than the Maryland average, including ≥1 schedule III or IV opioid prescriptions (30 per 100 000) and ≥1 schedule II opioid prescriptions (41 per 100 000).

## DISCUSSION

### Principal results

Patient matching within and across datasets is critical to constructing a complete picture of risk. Absent a common identifier that can be used to stitch together the data captured in fragmented datasets, other methods to match person-level data are being utilized in practice by state programs. Understanding how the matching method impacts the results of a risk model and prevalence of key risk indicators is important when making decisions about and responding to the opioid crisis. Although the results of this study cannot identify the absolute performance of models relative to a gold standard, the findings demonstrate how much variation is likely to exist with different matching approaches. Overall, this study found that prevalence of risk was highest in the approximate match population, but not uniformly, suggesting prevalence was also dependent on the type of risk indicator. Also, while the predictive model performance was similar across matching algorithms, the sensitivity and specificity varied, which has operational implications when designing interventions for the high-risk population identified.

The impact of matching is first reflected in the total number of individuals *within* the PDMP database after being processed by each algorithm. The approximate algorithm consolidated the population to the fewest identities and the exact + zip algorithm had the most. Although determining whether the algorithms correctly combined or did not combine identities was out of scope for this study, leaving some unknown number of false positives (ie, matching records of separate persons incorrectly together) and false negatives (ie, not matching the same person’s records together when they are the same person) within each population, the results demonstrate the relative impact that the patient matching approach can have on a study population, which can in turn impact measuring prevalence of a risk indicator. A nuance to evaluating the impact of patient matching on the prevalence of risk indicators highlighted in Table 4 is that it depends upon the nature of the measure itself. Conceptually, it may seem logical that better patient matching within a dataset will mean more individuals will meet high-risk thresholds due to the consolidation of the patient’s data used to compute the risk indicator. This was the case for MPE, in which the approximate algorithm resulted in the highest prevalence of individuals meeting the threshold. However, this was not the case with high MME, suggesting that in some cases when working with different matching methods, individuals could still meet certain PDMP thresholds even with lesser consolidation of an individual’s prescription history. Some individuals displaying high-risk patterns may be underestimated while other times the prevalence of a risk indicator in the population may be overestimated.

The greatest impact of the approach to patient matching was reflected in the cross-dataset matching between the PDMP data and the arrest and death data. As fewer individuals were matched with the arrests and deaths data, the death rates per 100 000 high-risk individuals are drastically deflated when using the exact + zip algorithm as compared with the approximate algorithm. If using the exact + zip algorithm alone to match individuals across datasets, the results of the analysis will have a noticeably lower prevalence of risk for the population. It may also miss some of the more high-risk individuals who have unstable housing or are purposefully evading detection by supplying variations of their demographics. The exact-basic algorithm is closer to the death rates demonstrated by the approximate linked population; however, it also has a lower prevalence of arrests and deaths. This suggests that using an approximate matching method may greatly improve finding high-risk populations, particularly when combining datasets, which should be explored in future research.

Model performance, defined in this study as how accurately the model was able to predict persons who fatally overdosed, did not vary greatly across the 3 matching algorithms; however, the sensitivity and specificity differed at the optimal risk model cutoff. The risk model for the approximate match dataset had a lower sensitivity and higher specificity, capturing fewer than half of the population identified as high risk as the model run on the exact match populations. Balancing sensitivity versus specificity is common practice with risk modeling and has practical implications for applied use of the model. When resources are scarce, such as the number of treatment beds, emphasis may want to be made on a higher specificity, where there is a lower likelihood of capturing individuals not at high risk, reserving beds for individuals at highest risk for future fatal opioid overdose. The higher specificity of the approximate match model demonstrated aptness toward interventions where scarce resources are being distributed, compared with the exact match populations. Alternatively, if the intervention allows for more latitude with who receives a service or resource, such as naloxone distribution, a higher sensitivity may be desired to cast a wider net, even if some individuals were incorrectly classified as high risk. The exact-basic matching may be suitable for these lower-cost, broad interventions based on the higher sensitivity at the risk score cutoff. It may also support the model serving as an analytic tool to understand population-level risk factors and effect sizes.

When weighing which algorithm to use in practice, the cost and complexity of establishing and maintaining the algorithm must be considered. The benefit of the exact matching is in its simplicity; no real long-term maintenance and quick to implement, which is why it is commonly used in practice today.^3^ Approximate match algorithms can be very complex and take in a larger number of data elements, leaving a higher opportunity for data quality to impact matching. Some publicly available approximate matching software exist, including Link Plus and The Link King, and several software companies sell approximate matching master data management solutions.^5^ Organizations leveraging approximate matching MPIs for operational purposes often have dedicated staff that monitor quality of the data used for matching, perform periodic clean-up to improve the matching rate, and assess algorithm weighting for continuous improvement. Because of this, MPIs are best for ongoing clinical or analytic purposes that require continuous use. If the matching is only needed periodically, it may not make financial sense to invest in a robust approximate match solution. This can be especially important for State government and PDMP programs that may not have sufficient access to expertise in deploying approximate match algorithms outside what a PDMP vendor may offer.

### Strengths and limitations

Given the nature of the datasets included in this study, completeness of patient identifiers was not a barrier to matching. The PDMP program requires basic patient information be supplied per state regulations and the arrest and death data ensures accurate patient information is captured as a matter of law. The approximate match algorithm contains a robust collection of demographics for Maryland residents over a long period of time, leading to improved matching. However, this strength is also a limitation. One of the benefits of the MPI (approximate match algorithm) is that it links records using historical and recent information. Replicating this process elsewhere may not be as successful if robust historical data is not present to improve the matching rate. This study was applied research based on tools available in practice within Maryland, thus future research should examine cleaner methods of matching that may be more applicable to other settings where similar data are available. Another notable limitation is that generally, literature evaluating matching algorithms perform “manual” reviews where a human assesses how often the algorithm properly classifies 2 individuals as a match or nonmatch.^6^^,^^23–25^ Although data were matched in an identifiable manner, this study only used deidentified linked records for analysis and consequently, this assessment could not be performed.

The timing of the data extracts for the approximate matching and exact matching was different, causing 2 issues. First, there was a slight difference in total number of prescriptions in the approximate versus exact match data extracts. The number of prescriptions was equalized between the 2 extracts prior to person-level analysis by creating a unique key based on multiple prescription-level attributes and removing prescriptions that were not present across all datasets. Second, the arrest data were linked using approximate matching within the dataset and delivered for analysis prior to this study. This resulted in only using the exact algorithm to match the arrest dataset with the PDMP and death datasets, but not within the arrest file. Additionally, only 2 exact match algorithms known to be used in practice today were analyzed, despite many algorithm options and variations being available for use, such as using partial name matches or other optimization techniques.^26^^,^^27^ The demographic elements used for the matching were largely influenced by the data availability in the datasets used for this study. There also is not a gold standard to which to compare the performance of the models in this study, which future work should address.

Future work should investigate the priori probability management to reduce selection biases, assess whether algorithms are creating more burden, and whether use of various algorithms result in potential adverse outcomes. Although many of the specific details are not generalizable beyond the particular datasets used in this study, the context of matching identities across disparate datasets is typical of one that confronts practitioners and researchers working with population-level datasets such as PDMP, and may prompt a more detailed exploration and assessment of existing and potential matching practices.^28–32^

## CONCLUSIONS

The understanding of risk indicator prevalence within and across disparate datasets varied across the matching approaches in use in applied settings. The model performance was not impacted by the matching approach; however, there are operational implications of using predictive models for an opioid intervention or program based on the balance of sensitivity and specificity. Moving forward, the frequency with which cross-sector datasets will be used to gain a comprehensive understanding of an individual’s risk of opioid-related overdose will only increase. Similar approaches will, and should, also be used to address other public health challenges. Additional studies that compare the performance of different matching algorithms in use by state-led programs at the identifiable patient-level to a gold standard should be performed. Further information on the impact of different matching methods, such as those explored in this study, will provide essential tools to state programs currently combining multiple datasets together to better identify individuals at high risk for opioid overdose death and design public health programs and interventions that benefit the community.

## FUNDING

This work was supported by the Bureau of Justice Assistance grant number 2015-PM-BX-K002. The Bureau of Justice Assistance is a component of the Department of Justice’s Office of Justice Programs, which also includes the Bureau of Justice Statistics, the National Institute of Justice, the Office of Juvenile Justice and Delinquency Prevention, the Office for Victims of Crime, and the SMART Office. Points of view or opinions in this document are those of the author and do not necessarily represent the official position or policies of the US Department of Justice, or the Maryland Department of Health. The study sponsors had no role in determining study design; data collection, analysis, or interpretation; writing the report; or the decision to submit the report for publication.

## AUTHOR CONTRIBUTIONS

LF contributed to the conception of the study, drafted the manuscript, identified, and developed key study variables, developed the analytic database, created and performed model analysis, and facilitated data linkage. JW, BS, and HK lead and oversaw the work, contributed to the conception of the study and revised the manuscript critically for important intellectual content.

## SUPPLEMENTARY MATERIAL

Supplementary material is available at *JAMIA Open* online.

## Supplementary Material

ooac020_Supplementary_Data
