# Supplementary material for: Comparing person-level matching algorithms to identify risk across disparate datasets among patients with a controlled substance prescription: retrospective analysis
Source: JAMIA Open. 2022 Mar 30;5(1):ooac020. doi: 10.1093/jamiaopen/ooac020 (PMC9097759; doi:10.1093/jamiaopen/ooac020)
Supplement: ooac020_Supplementary_Data [file ooac020_supplementary_data.docx]

**Supplementary File 1: Dataset Overlap Analysis**

| Approximate Matching Overlap | Exact-basic Matching Overlap |
| --- | --- |
| **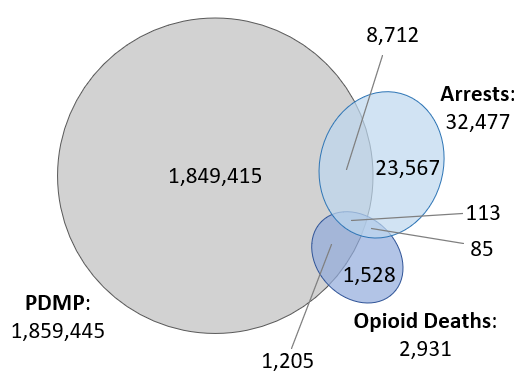** | **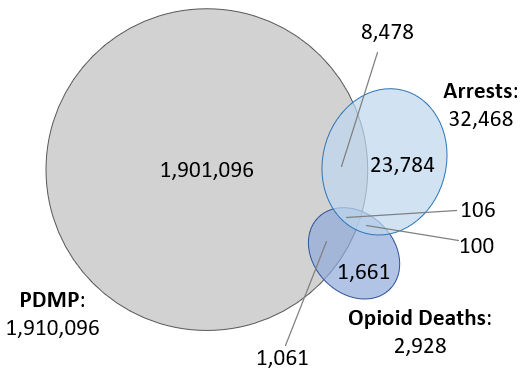** |
| Exact +zip Matching Overlap | Analytic Dataset |
| **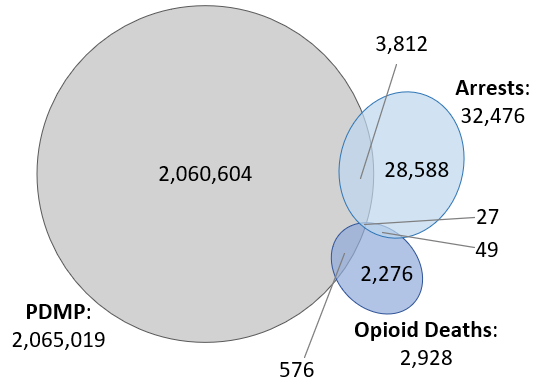** | **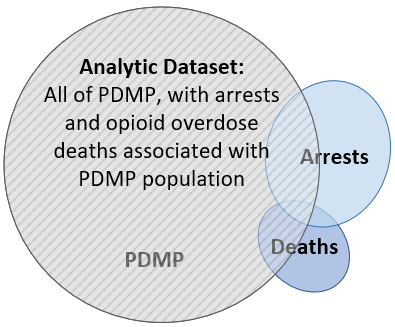** |

| **Within Dataset Matching*** | **Approximate** | **Exact-basic** | **Exact+zip** |
| --- | --- | --- | --- |
| PDMP Dataset (No. native identities: 3,304,446) | 1,859,445 | 1,910,741 | 2,065,019 |
| Arrest Dataset (No. native identities: 38,004) | 37,903 | 41,637 | 46,730 |
| OCME Dataset (No. native identities: 22,829) | 22,829 | 22,829 | 22,829 |
| **Across Dataset Matching**** | **Approximate** | **Exact-basic** | **Exact+zip** |
| PDMP Only | **1,849,415** | **1,901,096** | **2,060,604** |
| Arrests Only | 23,567 | 23,784 | 28,588 |
| Opioid Overdose Deaths Only | 1,528 | 1,661 | 2,276 |
| PDMP + Arrests | **8,712** | **8,478** | **3,812** |
| PDMP + Opioid Overdose Deaths | **1,205** | **1,061** | **576** |
| Arrests + Opioid Overdose Deaths | 85 | 100 | 49 |
| PDMP + Arrests + Opioid Overdose Deaths | **113** | **106** | **27** |
| **Bold** indicates the population used for this study's analysis.  * Within dataset matching timeframes and scope: PDMP=2015, Any property or drug arrest=2013-2016, Any investigated death=2012-2016.  ** Across dataset matching timeframes and scope: PDMP=2015, Any property or drug arrest=2013-2015, opioid-related overdose deaths=2015-2016  Abbreviations: PDMP-Prescription Drug Monitoring Program, OCME-Office of the Chief Medical Examiner | | | |

**Supplementary File 2: Approximate Matching Algorithm**

The approximate (probabilistic) matching algorithm can be broken into five basic functions:


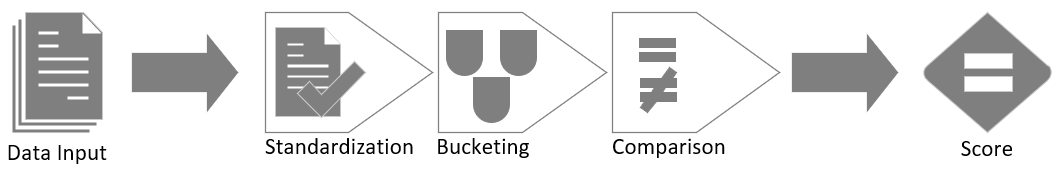


*Data Input*: The MPI algorithm uses demographic data elements normalized into different “attributes”. The algorithm attributes used to link individuals included name, date of birth, gender, address, phone number, and social security number. Each unique attribute was used to create a match score to represent the degree of certainty for an exact match.

*Standardization*: Standardization represents the conversion of the data into its simplest form to allow for easy comparison. The standardization steps used included removing false or anonymous values, such as phone numbers entered as (000) 000-0000, removing any special characters and applying truncations, such as (123) 456-7890 to 1234567890, and converting the name to all upper case.^34^ Two types of address standardization arguments were employed: (1) postal codes patterning, and (2) type of words or characters used for unit information (e.g., floor, suite, unit).

*Bucketing*: Bucketing organizes common data values together using single or multiple attributes to create unique combinations that are more easily recalled during searching and matching. Theoretically, each identity needs to be compared with every previously processed identity, which would be far too consuming without a mechanism to limit it only logical comparisons. The bucketing processes employed by the MPI used in this study included Name (First Name + Last Name), Name Phonetic + DOB, Name Phonetic + Zip Code, SSN, Phone, Zip Code, MRN, plus some special attributes specific to Maryland stakeholders.

*Comparison*: The individuals sharing buckets together are then compared and scored using predefined probabilistically generated weights. Each comparison function for the different attributes generates a score that can be positive or negative. Full points were awarded for exact matches and partial points were given for common but minor data discrepancies, such as the use of nicknames, misspellings, and transposed dates, names, and numbers. The comparison function incorporates various approaches to processing information that inherently have data quality challenges:

- Initials and full word comparison: match on initials or full word are assessed and scored.
- Enhanced soundex: words with similar phonetic sounds receive a higher score
- Frequency indexing: common words and names yield lower scores; uncommon words and names yield higher scores.
- Nickname tables: tables that equate formal and informal names and cross-compare the exact and phonetic names.
- Edit distance calculations: the number of changes needed for two values to be equivalent, the lower the number of changes the more likely the records are a match. Prefix and compound comparisons are also employed where matches may be missed by the edit-distance calculation, for example, Belair and Bel Air would be compared.
- Acronym comparison: for example, D.C. would be compared with District of Columbia.
- Attribute weights: for example, phone number receives a higher weight than gender.
- Historical values in matching: the use of previous addresses or names (maiden names) as part of the matching technology improves the matching capability.
- False positive filter: applies deterministic logic to specific false positive matches and uses the result to apply a penalty score.

*Score*: The final score is a sum of scores from the comparison functions in the algorithm and reflects the degree of match certainty between the two individuals being compared. The patient was a “match” if above the CRISP-defined threshold (score≥13.1), “potential match” if the individuals are close but not strong enough to be linked together (score between 13.1 and 10.2), or “not matched” (score≥10.1). The attributes configured for comparison and MPI weight distributions include:

| **Attribute** | **Max Weight** | **Min Weight** |
| --- | --- | --- |
| **Name / Previous Name (1 attribute)** | 4.08 (max 7.00) | Invalid: -2.35; Other: -1.45 |
| **Date of Birth (1 attribute)** | 4.75 | -3.92 |
| **Gender (1 attribute)** | M:.23; F:.38; O:.23 | -2.03 |
| **SSN – Full (1 attribute)** | 3.66 | -5.37 |
| **SSN - Last four (1 attribute)** | 1.85 | -1.00 |
| **Patient Address and Phone Combinations (2 attributes)** |  |  |
| Patient Address: missing, Phone: missing | 0 | N/A |
| Patient Address: match, Phone: missing | 5.01 | N/A |
| Patient Address: missing, Phone: match | 4.79 | N/A |
| Patient Address: match, Phone: match | 5.24 | N/A |
| Patient Address: no match, Phone: missing | 4.03 | -2.83 |
| Patient Address: missing, Phone: no match | 4.75 | -3.61 |
| Patient Address: no match, Phone: no match | 4.95 | -2.19 |
| **False Positive filter (4 attributes)** |  |  |
| Name: match, Gender: missing, DOB: apart >15 years, SSN: missing |  | -3.00 |
| Name: match, Gender: missing, DOB: apart >15 years, SSN: not missing |  | -5.00 |
| Name: partial match, Gender: missing, DOB: apart >15 years, SSN: missing |  | -3.00 |
| Name: partial match, Gender: missing, DOB: apart >15 years, SSN: not missing |  | -5.00 |
| Name: no match, Gender: missing, DOB: match |  | -5.00 |
| Name: no match, Gender: missing, DOB: apart >15 years |  | -5.00 |
| Name: match, Gender: match, DOB: apart >15 years, SSN: missing |  | -3.00 |
| Name: match, Gender: match, DOB: apart >15 years, SSN: not missing |  | -5.00 |
| Name: partial match, Gender: match, DOB: match, SSN: missing |  | -2.00 |
| Name: partial match, Gender: match, DOB: match, SSN: match |  | -2.00 |
| Name: partial match, Gender: match, DOB: match, SSN: edit distance | -2.50 | -5.00 |
| Name: partial match, Gender: match, DOB: apart >15 years, SSN: missing |  | -3.00 |
| Name: partial match, Gender: match, DOB: apart >15 years, SSN: not missing |  | -5.00 |
| Name: no match, Gender: match, DOB: match, SSN: missing |  | -4.00 |
| Name: no match, Gender: match, DOB: match, SSN: match |  | -3.50 |
| Name: no match, Gender: match, DOB: match, SSN: edit distance | -4.00 | -5.00 |
| Name: no match, Gender: match, DOB: apart >15 years |  | -5.00 |
| Name: match, Gender: not match, DOB: apart >15 years, SSN: missing |  | -3.00 |
| Name: match, Gender: not match, DOB: apart >15 years, SSN: not missing |  | -5.00 |
| Name: partial match, Gender: no match, DOB: match, SSN: missing or no match |  | -2.50 |
| Name: partial match, Gender: no match, DOB: apart >15 years, SSN: missing |  | -3.00 |
| Name: partial match, Gender: no match, DOB: apart >15 years, SSN: not missing |  | -5.00 |
| Name: no match, Gender: no match, DOB: match |  | -5.00 |
| Name: not match, Gender: not match, DOB: apart >15 years |  | -5.00 |

An example scoring of two individual’s test demographics (fake patient) processed through the operational MPI’s probabilistic algorithm is represented below:

| **Comparison** | **Grape, Gilbert, PDMP:9900991** | **Gilbert, Grape, DPSCS: 55324343** | **Weight** |
| --- | --- | --- | --- |
| XNM | GRAPE\|GILBERT\| | GILBERT\|GRAPE\| | 6.84 |
| Date of Birth | 19840101 | 19840101 | 4.75 |
| Gender | F | F | 0.23 |
| Address | 4145\|EARL\|C\|ADKINS\|DR\|RIVER\|WV\|26000 | 4145\|EARL\|C\|ADKINS\|DR\|RIVER\|WV\|26000 | 5.01 |
| Phone | 301-222-2999 |  |  |
| SSN | X16326289 | X214024632 | -2.66 |
| False Positive Filter | 19840101 | 19840101 | 0 |
| **Total Match Score** | | | **14.17** |

### **Supplementary File 3: Exact Algorithm Matching**

Two exact algorithms were applied to each dataset, starting with PDMP data.

The matching process consisted of the following steps:

1. Start with an empty master patient list.
2. Begin processing the identities in each file using the defined algorithm (i.e. first name, last name, gender, date of birth), comparing each identity in the database to the identities in the master patient list.
   1. If any exact matches occurred between the master list of the unique identities and the dataset, the identity in the dataset was assigned the master exact ID in the master list.
   2. If no match exists, the identity is added to the master patient list and assigned its own unique master exact ID (i.e., this applies to the very first identity processed – the identity was added since there was no exact match to be made with an empty list)
3. Steps 2a and 2b were repeated for the remaining datasets, one dataset at a time.

The steps outlined above were repeated for each dataset. Because the master list of identities was for all unique individuals, if there were multiple records within a single database with matching demographics, the master exact ID would be applied across all records, therefore matching records within a single database as well (not just across). For transaction-level databases (PDMP and DPSCS), identities that had the same dataset-defined patient identifier within a single database was checked to ensure the same master exact ID was applied. Any identities in the dataset that did not have an assigned database-level unique patient identifier were removed before the processing began.

### **Supplementary File 4: Quality of Matching Fields**

| **Dataset** | **# Native Dataset Identities** | **First Name** | **Last Name** | **Sex** | **DOB** | **Address** | **City** | **Zip** | **SSN** |
| --- | --- | --- | --- | --- | --- | --- | --- | --- | --- |
| PDMP ^b^ | 3,304,446 | 3,304,440 (100) | 3,304,363 (100) | 3,304,000 (100) | 3,303,391 (100) | 3,303,546 (100) | 3,304,171 (100) | 3,303,515 (100) | 0 (0.0) |
| Arrests ^c^ | 118,218 | 118,218 (100) | 118,218 (100) | 118,218 (100) | 118,218 (100) | 114,311 (96.7) | 114,087 (96.5) | 113,806 (96.3) | 72,093 (61.0) |
| Deaths ^d^ | 22,829 | 22,828 (100) | 22,827 (100) | 22,791 (99.8) | 22,704 (99.8) | 21,888 (95.9) | 21,747 (95.3) | 21,404 (93.8) | 0 (0.0) |
| Abbrev.: PDMP=Prescription Drug Monitoring Program, DOB=Date of Birth, SSN=Social Security Number.  ^a^ Demographic data were used by the health information exchange to match identities within and across datasets prior to removing patient identifiers from final analytic dataset. This table reflects the completeness of the demographic data (based on missing values) used for matching. All demographic fields were utilized for the approximate matching. Exact matching only relied upon first name, last name, sex, date of birth, and zip.  ^b^ PDMP data consists of all controlled substance prescriptions dispensed in 2015.  ^c^ Arrest data consists drug and property arrests from 2013-2016 (narrowed to arrests in 2013-2015 for analysis).  ^d^ Death data consists of outcome of all investigated deaths from 2012-2016 (narrowed to opioid-related overdose deaths in 2015-2016 for analysis). | | | | | | | | | |

### **Supplementary File 5: Predictive Model Classification Tables for Matching Algorithms**

| **Probabilistic Algorithm - validation cohort (n = 661,451)** | | | | | | | | | | | | | | | | | | | | | | | | | | | | |
| --- | --- | --- | --- | --- | --- | --- | --- | --- | --- | --- | --- | --- | --- | --- | --- | --- | --- | --- | --- | --- | --- | --- | --- | --- | --- | --- | --- | --- |
| Model Risk Score Cutoff | | | Sens. | | Spec. | | Sens. + Spec. | | | | PPV | | NPV | | | Acc. | | # high-risk patients | | | | validation cohort % | | # Deaths high-risk pts | | Deaths per 1k high-risk pts | |  |
| 0.00025 | | 95.51 | | | 38.15 | | | 133.66 | | 0.12 | | | | 99.99 | | | 28.19 | | | 409782 | | | 62.0% | | 511 | | 1.25 | |
| 0.0005 | | 87.87 | | | 61.37 | | | 149.24 | | 0.18 | | | | 99.98 | | | 61.40 | | | 256,047 | | | 38.7% | | 471 | | 1.84 | |
| **0.0010** | | **67.54** | | | **84.29** | | | **151.83** | | **0.35** | | | | **99.97** | | | **84.28** | | | **104,293** | | | **15.8%** | | **362** | | **3.47** | |
| 0.0015 | | 56.16 | | | 90.78 | | | 146.94 | | 0.49 | | | | 99.96 | | | 90.76 | | | 61,286 | | | 9.3% | | 301 | | 4.91 | |
| 0.0020 | | 48.88 | | | 93.79 | | | 142.67 | | 0.63 | | | | 99.96 | | | 93.76 | | | 41,325 | | | 6.2% | | 262 | | 6.34 | |
| 0.0025 | | 44.59 | | | 95.11 | | | 139.70 | | 0.73 | | | | 99.95 | | | 95.07 | | | 32,564 | | | 4.9% | | 239 | | 7.34 | |
| 0.0030 | | 36.75 | | | 96.45 | | | 133.20 | | 0.83 | | | | 99.95 | | | 96.40 | | | 23,695 | | | 3.6% | | 197 | | 8.31 | |
| 0.0035 | | 33.21 | | | 96.99 | | | 130.20 | | 0.88 | | | | 99.94 | | | 96.93 | | | 20,122 | | | 3.0% | | 178 | | 8.85 | |
| 0.0040 | | 29.29 | | | 97.53 | | | 126.82 | | 0.95 | | | | 99.94 | | | 97.47 | | | 16,506 | | | 2.5% | | 157 | | 9.51 | |
| 0.0045 | | 25.75 | | | 97.86 | | | 123.61 | | 0.96 | | | | 99.94 | | | 97.80 | | | 14,304 | | | 2.2% | | 138 | | 9.65 | |
| 0.0050 | | 24.44 | | | 98.07 | | | 122.51 | | 1.02 | | | | 99.94 | | | 98.01 | | | 12,897 | | | 1.9% | | 131 | | 10.16 | |
| 0.0055 | | 22.39 | | | 98.32 | | | 120.71 | | 1.07 | | | | 99.94 | | | 98.25 | | | 11,263 | | | 1.7% | | 120 | | 10.65 | |
| 0.0060 | | 19.96 | | | 98.52 | | | 118.48 | | 1.08 | | | | 99.93 | | | 98.46 | | | 9,886 | | | 1.5% | | 107 | | 10.82 | |
| 0.0065 | | 16.98 | | | 98.85 | | | 115.83 | | 1.18 | | | | 99.93 | | | 98.78 | | | 7,721 | | | 1.2% | | 91 | | 11.79 | |
| 0.0070 | | 16.04 | | | 98.94 | | | 114.98 | | 1.22 | | | | 99.93 | | | 98.88 | | | 7,072 | | | 1.1% | | 86 | | 12.16 | |
| 0.0075 | | 15.30 | | | 99.02 | | | 114.32 | | 1.25 | | | | 99.93 | | | 98.96 | | | 6,541 | | | 1.0% | | 82 | | 12.54 | |
| 0.0080 | | 14.37 | | | 99.08 | | | 113.45 | | 1.26 | | | | 99.93 | | | 99.02 | | | 6,133 | | | 0.9% | | 77 | | 12.56 | |
| 0.0085 | | 12.87 | | | 99.18 | | | 112.05 | | 1.25 | | | | 99.93 | | | 99.11 | | | 5,518 | | | 0.8% | | 69 | | 12.50 | |
| 0.0090 | | 11.75 | | | 99.26 | | | 111.01 | | 1.27 | | | | 99.93 | | | 99.19 | | | 4,971 | | | 0.8% | | 63 | | 12.67 | |
| 0.0095 | | 11.01 | | | 99.32 | | | 110.33 | | 1.29 | | | | 99.93 | | | 99.25 | | | 4,570 | | | 0.7% | | 59 | | 12.91 | |
| Abbreviations: Acc.=accuracy, NPV=negative predictive value, PPV=positive predictive value, Sens.=sensitivity, Spec.=specificity, pts=patients | | | | | | | | | | | | | | | | | | | | | | | | | | | | |
| **Exact-basic Algorithm - validation cohort (n = 679,925)** | | | | | | | | | | | | | | | | | | | | | | | | | | | | |
| Model Risk Score Cutoff | | | Sens. | | Spec. | | Sens. + Spec. | | | | PPV | | NPV | | | Acc. | | # high-risk patients | | | | validation cohort % | | # Deaths high-risk pts | | Deaths per 1k high-risk pts | |  |
| 0.00025 | 94.97 | | | 45.54 | | 140.51 | | | 0.11 | | | 99.99 | | | 44.58 | | | | 377,229 | | 55.48 | | | 415 | | | 1.10 | |
| **0.0005** | **87.47** | | | **66.26** | | **153.73** | | | **0.17** | | | **99.99** | | | **66.27** | | | | **229,646** | | **33.78** | | | **384** | | | **1.67** | |
| 0.0010 | 66.74 | | | 85.99 | | 152.73 | | | 0.31 | | | 99.98 | | | 85.98 | | | | 95,460 | | 14.04 | | | 293 | | | 3.07 | |
| 0.0015 | 57.63 | | | 91.03 | | 148.66 | | | 0.41 | | | 99.97 | | | 91.01 | | | | 61,202 | | 9.00 | | | 253 | | | 4.13 | |
| 0.0020 | 47.15 | | | 94.30 | | 141.45 | | | 0.53 | | | 99.96 | | | 94.27 | | | | 38,923 | | 5.72 | | | 207 | | | 5.32 | |
| 0.0025 | 39.18 | | | 95.79 | | 134.97 | | | 0.60 | | | 99.96 | | | 95.75 | | | | 28,805 | | 4.24 | | | 172 | | | 5.97 | |
| 0.0030 | 35.99 | | | 96.77 | | 132.76 | | | 0.71 | | | 99.96 | | | 96.73 | | | | 22,120 | | 3.25 | | | 158 | | | 7.14 | |
| 0.0035 | 33.03 | | | 97.37 | | 130.40 | | | 0.80 | | | 99.96 | | | 97.32 | | | | 18,042 | | 2.65 | | | 145 | | | 8.04 | |
| 0.0040 | 31.44 | | | 97.70 | | 129.14 | | | 0.88 | | | 99.95 | | | 97.66 | | | | 15,759 | | 2.32 | | | 138 | | | 8.76 | |
| 0.0045 | 26.88 | | | 98.00 | | 124.88 | | | 0.86 | | | 99.95 | | | 97.96 | | | | 13,680 | | 2.01 | | | 118 | | | 8.63 | |
| 0.0050 | 23.69 | | | 98.44 | | 122.13 | | | 0.97 | | | 99.95 | | | 98.39 | | | | 10,684 | | 1.57 | | | 104 | | | 9.73 | |
| 0.0055 | 22.32 | | | 98.60 | | 120.92 | | | 1.02 | | | 99.95 | | | 98.55 | | | | 9,600 | | 1.41 | | | 98 | | | 10.21 | |
| 0.0060 | 19.13 | | | 98.80 | | 117.93 | | | 1.02 | | | 99.95 | | | 98.75 | | | | 8,241 | | 1.21 | | | 84 | | | 10.19 | |
| 0.0065 | 15.95 | | | 98.96 | | 114.91 | | | 0.98 | | | 99.95 | | | 98.90 | | | | 7,164 | | 1.05 | | | 70 | | | 9.77 | |
| 0.0070 | 15.26 | | | 99.06 | | 114.32 | | | 1.04 | | | 99.94 | | | 99.01 | | | | 6,427 | | 0.95 | | | 67 | | | 10.42 | |
| 0.0075 | 13.67 | | | 99.17 | | 112.84 | | | 1.06 | | | 99.94 | | | 99.12 | | | | 5,676 | | 0.83 | | | 60 | | | 10.57 | |
| 0.0080 | 13.21 | | | 99.24 | | 112.45 | | | 1.12 | | | 99.94 | | | 99.19 | | | | 5,199 | | 0.76 | | | 58 | | | 11.16 | |
| 0.0085 | 12.76 | | | 99.30 | | 112.06 | | | 1.17 | | | 99.94 | | | 99.25 | | | | 4,788 | | 0.70 | | | 56 | | | 11.70 | |
| 0.0090 | 12.30 | | | 99.36 | | 111.66 | | | 1.22 | | | 99.94 | | | 99.30 | | | | 4,416 | | 0.65 | | | 54 | | | 12.23 | |
| 0.0095 | 12.07 | | | 99.41 | | 111.48 | | | 1.31 | | | 99.94 | | | 99.36 | | | | 4,051 | | 0.60 | | | 53 | | | 13.08 | |
| Abbreviations: Acc.=accuracy, NPV=negative predictive value, PPV=positive predictive value, Sens.=sensitivity, Spec.=specificity, pts=patients | | | | | | | | | | | | | | | | | | | | | | | | | | | | |
| **Exact+zip Algorithm - validation cohort (n = 727,565)** | | | | | | | | | | | | | | | | | | | | | | | | | | | |  |
| Model Risk Score Cutoff | | | Sens. | | Spec. | | Sens. + Spec. | | | | PPV | | NPV | | | Acc. | | # high-risk patients | | | | validation cohort % | | # Deaths high-risk pts | | Deaths per 1k high-risk pts | |  |
| **0.00025** | | | **85.53** | | **62.17** | | 147.70 | | | | **0.07** | | **99.99** | | | **62.18** | | **275,352** | | | | **37.85** | | **195** | | **0.71** | |  |
| 0.0005 | | | 60.96 | | 84.37 | | 145.33 | | | | 0.12 | | 99.99 | | | 84.37 | | 113,791 | | | | 15.64 | | 139 | | 1.22 | |  |
| 0.0010 | | | 42.54 | | 94.30 | | 136.84 | | | | 0.23 | | 99.98 | | | 94.29 | | 41,531 | | | | 5.71 | | 97 | | 2.34 | |  |
| 0.0015 | | | 34.21 | | 96.98 | | 131.19 | | | | 0.35 | | 99.98 | | | 96.96 | | 22,074 | | | | 3.03 | | 78 | | 3.53 | |  |
| 0.0020 | | | 26.32 | | 98.00 | | 124.32 | | | | 0.41 | | 99.98 | | | 97.97 | | 14,629 | | | | 2.01 | | 60 | | 4.10 | |  |
| 0.0025 | | | 22.37 | | 98.55 | | 120.92 | | | | 0.48 | | 99.98 | | | 98.53 | | 10,580 | | | | 1.45 | | 51 | | 4.82 | |  |
| 0.0030 | | | 17.98 | | 98.94 | | 116.92 | | | | 0.53 | | 99.97 | | | 99.92 | | 7,727 | | | | 1.06 | | 41 | | 5.31 | |  |
| 0.0035 | | | 14.91 | | 99.22 | | 114.13 | | | | 0.60 | | 99.97 | | | 99.19 | | 5,713 | | | | 0.79 | | 34 | | 5.95 | |  |
| 0.0040 | | | 10.53 | | 99.38 | | 109.91 | | | | 0.53 | | 99.97 | | | 99.35 | | 4,515 | | | | 0.62 | | 24 | | 5.32 | |  |
| 0.0045 | | | 9.21 | | 99.49 | | 108.70 | | | | 0.56 | | 99.97 | | | 99.46 | | 3,761 | | | | 0.52 | | 21 | | 5.58 | |  |
| 0.0050 | | | 8.77 | | 99.58 | | 108.35 | | | | 0.65 | | 99.97 | | | 99.55 | | 3,075 | | | | 0.42 | | 20 | | 6.50 | |  |
| 0.0055 | | | 7.46 | | 99.63 | | 107.09 | | | | 0.63 | | 99.97 | | | 99.60 | | 2,686 | | | | 0.37 | | 17 | | 6.33 | |  |
| 0.0060 | | | 7.46 | | 99.69 | | 107.15 | | | | 0.31 | | 99.97 | | | 99.66 | | 2,301 | | | | 0.32 | | 17 | | 7.39 | |  |
| 0.0065 | | | 6.14 | | 99.72 | | 105.86 | | | | 0.69 | | 99.97 | | | 99.69 | | 2,041 | | | | 0.28 | | 14 | | 6.86 | |  |
| 0.0070 | | | 5.26 | | 99.77 | | 105.03 | | | | 0.73 | | 99.97 | | | 99.74 | | 1,654 | | | | 0.23 | | 12 | | 7.26 | |  |
| 0.0075 | | | 3.95 | | 99.80 | | 103.75 | | | | 0.61 | | 99.97 | | | 99.77 | | 1,483 | | | | 0.20 | | 9 | | 6.07 | |  |
| 0.0080 | | | 3.95 | | 99.81 | | 103.76 | | | | 0.66 | | 99.97 | | | 99.78 | | 1,358 | | | | 0.19 | | 9 | | 6.63 | |  |
| 0.0085 | | | 3.51 | | 99.83 | | 103.34 | | | | 0.65 | | 99.97 | | | 99.80 | | 1,225 | | | | 0.17 | | 8 | | 6.53 | |  |
| 0.0090 | | | 3.51 | | 99.85 | | 103.36 | | | | 0.74 | | 99.97 | | | 99.82 | | 1,082 | | | | 0.15 | | 8 | | 7.39 | |  |
| 0.0095 | | | 3.07 | | 99.86 | | 102.93 | | | | 0.70 | | 99.97 | | | 99.83 | | 993 | | | | 0.14 | | 7 | | 7.05 | |  |
| Abbreviations: Acc.=accuracy, NPV=negative predictive value, PPV=positive predictive value, Sens.=sensitivity, Spec.=specificity, pts=patients | | | | | | | | | | | | | | | | | | | | | | | | | | | |  |
